# The Student at the Apex

**Published:** 1893-09-30

**Authors:** 


### The Hospital

An Institutional Journal of

**Science, Medicine, Nursing, and Philanthropy.**

*For Hospitals, Asylums, Infirmaries, Dispensaries, Medical Practitioners, Students, and Nurses.*

Vol. XIV.—No. 366.] [Sept. 30, 1893.

### The Student at the Apex.

Among medical students, as among other classes of people, there are those who think and those who do not think. To the thinker, medicine and its accompanying sciences constitute one of the happiest and most intellectual of callings; whereas to the non-thinker, nothing, one would imagine, can be more utterly wearisome and unreal than the daily routine of medical practice. One of the first things the thinking medical student has to try to realise is that medicine, which was once at the base, is now at the apex of the pyramid of human knowledge. A great part of the new knowledge which science has brought into service is physiological, or to use a more truly comprehensive and descriptive word, biological. Biology is one of the conquests of medicine, and serves the purposes of scientific medicine in a thousand ways.

The consciousness of being at the apex of the pyramid of knowledge, in a time of extraordinary intellectual activity, is a sobering and an arresting consciousness. He who has knowledge points the way to him who has none, as the seeing man leads the blind. At the present stage of the world's progress the man who knows is the man who leads—who leads and rules. The fact to be impressed upon and thoroughly worked into every intelligent medical student's consciousness is that he is destined to acquire a vast amount of scientific knowledge of which the mass of mankind are destined to remain ignorant; and to him the ignorant world will constantly look for honest and capable leadership.

It was a sobering moment when the French soldiers of 1870 found themselves fairly in the midst of the populations of Germany, and grappling with her terrible armies. It would be a still more sobering moment if the armies of the two countries, with their larger numbers and their newer and deadlier weapons, should meet in dread conflict to-day: sobering for the private soldier, more sobering for the leaders and generals, sobering most of all for the commander-in-chief. Similarly it is profoundly sobering to the medical student and practitioner of broad and strong intellectual capacity to know that he is destined to be a leader, perhaps a commander-in-chief in the world's armies of expanding knowledge and practical affairs. What an age thinks that it will do. It may be an age of high thinking, and then it will be an age of high and noble achievement. It may be an age of low, mean, sordid, or craven thinking, and then it will be an age of base and unworthy deeds. The age will think as its leaders think, and the leaders of our own age are and will be, among others, biologists, physiologists, and physicians. This, we say, is an arresting thought to the medical student who is capable of competent thinking.

For the brave and resolute man responsibility has no terrors. It is not a burden which crushes, but a stimulus which rouses and invigorates him. The spirits of the medical student rise high as he realises that it is his destiny to be, not a mere journeyman in the great world's affairs, but a man amongst men, a thinker amongst thinkers, a leader amongst those who know and lead. This courageous and expanding attitude of mind is the attitude of all others which sees truth clearly, and sees it whole; which does not merely touch the fringe of knowledge with the timidity of an untaught slave, but which conquers it and takes possession like a strong and victorious master.

Every new day the medical student realizes more decidedly and clearly that he entered upon a prodigious task when he chose the conquest of all the fields of knowledge which medicine includes for his life work; and every new day the idler or the timid man becomes more prostrate and helpless beneath the accumulating load. But it is not so with the stronger spirit which sees the "joy of knowledge" that is set before him. The hope of being a man whose knowledge the world will respect, whose character it will trust, and whose aid it will seek in the new, and as yet almost unimagined struggles of the near future, is an inspiration which removes mountains, and vanquishes difficulties as the summer sun devours the morning mist.

The medical student cannot play the leader's or the teacher's part to any great extent whilst he is still a student. His knowledge is too green and raw, too newly acquired for that. It is like a company of recruits; he has enlisted it, and it is his own; but it requires, like the recruits, to be drilled and shaken down, and ordered, and disciplined in his mind. The clever student or newly admitted practitioner is apt to claim his command too soon; and he is minded to brush away his seniors out of his path as if they were mere obstructions and dead weight. He will smile at himself when he is a little older. He will recollect then, what he is apt to forget in the first crude state of his knowledge, that many of those seniors he now despises were clever students like himself in the long ago, and that now they are clever men, albeit somewhat sobered and subdued by a knowledge and experience a hundred times greater than that which the cleverest student can possess. The student of to-day will be sober in his turn

But though there can be no actual taking of command in the student days, there should be constantly present the consciousness that the days of riper knowledge and of full leadership will surely come; and there must be constantly present the determination to prepare for those days by knowledge of the widest, deepest, and most philosophically apprehended nature, which can be attained to. In time of peace prepare for war; and in the days of studentship every man should prepare himself as if he were to occupy the highest, most responsible, and most trusted position in the medical profession. He who fits himself for that position will be fit for any other; and, therefore, for the very position which destiny shall finally call upon him to accept as his own.

No man who stands at the apex of human knowledge can divest himself of profound responsibilities. The position is proud and high, and the obligations are correspondingly high and hourable. The position is also a novel one for the medical man. A new position demands a new man and new capacities. The medical student should rise to this new level. The old-fashioned student is dead and buried, and the old-fashioned practitioner is dying. The new man must have a new mind. He must kill evil traditions, both as a student and as a practitioner. He must, both as a student and a practitioner, "cut prejudice against the grain," as Tennyson said. Medical prejudice has a very tough grain, and takes a great deal of cutting. Injurious prejudices, like microbes, die in the sunlight. The student must open wide the windows of his mind, and let the sun of pure knowledge shine in unchecked. It will be intellectual ruin and death to him if he does not. He must claim, and defend, his intellectual freedom to the very death.

The career which lies before absolutely free, unprejudiced, and open medical minds is great almost beyond compare. Scientific medicine is destined to lead the world in the physical sphere. It is the high duty of medical men and medical students to see that the world is rationally guided. He who aspires to leadership must have courage. Of old medicine was timid. Timidity is one of its most conspicuous weaknesses even yet. In the things of science the doctor is bold; in the greater and nobler things of humanity he is often an unhappy compound of timidity and feebleness. This state of things is to be ended, or at least mended; and we who are in the midst of the battle look to the free and unprejudiced spirits of youth to present a braver, larger, nobler, and more truly human medicine to the world than has been presented in the dark or but feebly-illuminated ages which have gone before.

